# Late diagnosis of a congenitally corrected transposition of the great arteries discovered at pacemaker implantation in a patient previously diagnosed with dextrocardia and situs solitus

**DOI:** 10.1002/ccr3.1541

**Published:** 2018-04-22

**Authors:** Andreea Vasiliu, Stéphanie Seldrum, Michaël Dupont, Fabien Dormal, Dominique Blommaert, Luc De Roy

**Affiliations:** ^1^ Université Catholique de Louvain CHU UCL Namur Service de Cardiologie 1 Av Dr G Therasse 5530 Yvoir Belgium; ^2^ Université Catholique de Louvain CHU UCL Namur Service de Radiologie 1 Av Dr G Therasse 5530 Yvoir Belgium

**Keywords:** CCTGA, dextrocardia, pacemaker

## Abstract

Congenitally corrected transposition of the great arteries (CCTGA) should not be missed in patients with dextrocardia and situs solitus. We report a case of a 56‐year‐old man with late diagnosis of CCTGA after ventricular lead replacement. Free LV wall pacing may be favorable in these patients so to prevent deterioration of the systemic RV function.

## Introduction

Congenitally corrected transposition of the great arteries (CCTGA) is a rare cardiac condition representing less than 1% of cases of congenital heart diseases and is often associated with other cardiac malformations, such as perimembranous ventricular septal defects (70%), pulmonary or subpulmonary stenosis (40%), and abnormalities of the systemic atrioventricular (AV) valve (90% of patients have “Ebstein”‐like malformations) 1.

Only 1% of cases is uncomplicated, appearing as isolated CCTGA, and can be asymptomatic until late adulthood. Progressive systemic (tricuspid) AV valve regurgitation and systemic (right) ventricular dysfunction tend to occur from the fourth decade onwards, whereas atrial arrhythmias are more common from the fifth decade onwards, ventricular rhythm disturbances being rarely described in the natural history of CCTGA 2.

Due to an abnormal position of the AV node 3, these patients also tend to develop atrioventricular (AV) conduction problems. In fact, the probability of a spontaneous complete heart block increases linearly with age at a rate of approximately 2% per year 4; hence, permanent pacemaker implantation is often required.

Some patients also have dextrocardia, that is, most of the cardiac mass positioned in the right hemithorax and a base to apex axis pointing to the right 5. The literature regarding pacemaker implantation in patients with dextrocardia and CCTGA is scarce and is limited to a few case reports 5, 6.

## Case Description

An asymptomatic 56‐year‐old man attended our institution for a routine follow‐up of his pacemaker, implanted 20 years earlier for syncope and paroxysmal second‐degree AV block with a 2:1 conduction. At that time, an electrophysiological study was performed showing an AH interval of 114 ms and a HV interval of 42 ms for conducted beats and confirming the diagnosis of suprahisian block. One‐to‐one AV conduction resumed during stress test. The patient had a prior diagnosis of dextrocardia and situs solitus.

The pacemaker was programmed in a VDD pacing mode (ventricular pacing with atrial tracking), with a unipolar ventricular detection and pacing mode, and a bipolar atrial sensing feature. The pacemaker's pulse generator had previously been replaced twice.

**Figure 1:**
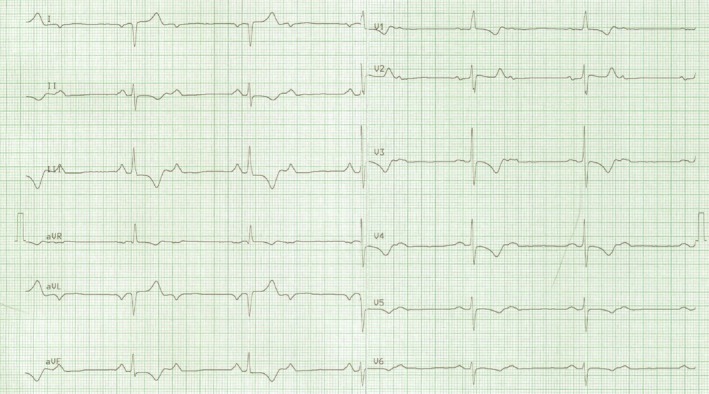
An ECG showing a second‐degree AV block with a ventricular rate of 36 bpm. P waves are negative in lead I and aVL, suggestive of dextrocardia. The absence of Q waves in V5–V6 is in accordance with CCTGA.

The spontaneous rhythm without pacemaker intervention showed a second‐degree AV block with a ventricular rate of 36 bpm and typical features of dextrocardia: a negative P wave in lead I, and an R wave amplitude decreasing from V3 to V6 (Fig. 1).

During pacemaker control, we observed a high impedance of 1460 ohm and pacing failure, with an absence of spikes even after programmed high pacing output energy of 6.5 V/1 ms pulse width. There was a complete sensing failure, which suggested a loss of lead integrity.

A posteroanterior X‐ray was compatible with dextrocardia, with the pacemaker generator positioned in the right pectoral area and a clear lead fracture, probably at the level of the AV valve. We decided to replace the pacemaker with a dual chamber pulse generator and new atrial and ventricular leads. An active fixation ventricular lead was thus positioned in the ventricle, near the tip of the older lead and the atrial lead in the right atrial appendage. Satisfactory pacing and sensing parameters were obtained for both.

**Figure 2:**
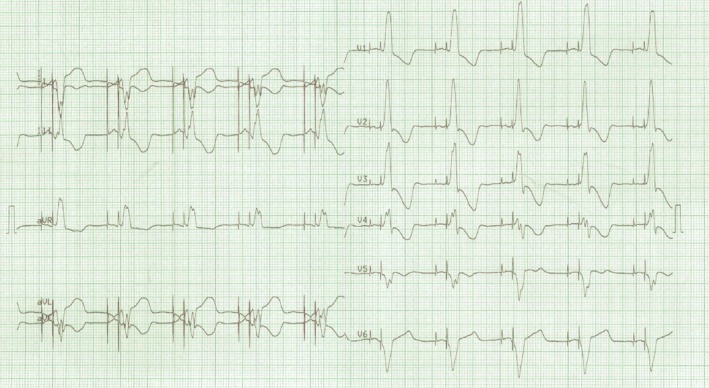
An ECG revealing ventricular paced complexes with a monophasic R wave in lead V1 and a right axis deviation.

**Figure 3:**
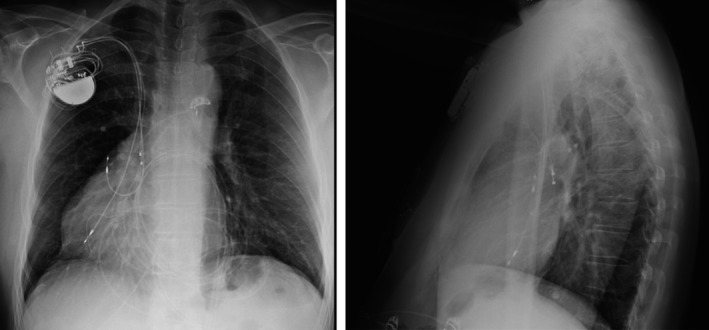
An X‐ray revealing a normal position of the right atrial lead with the new ventricular lead orientated laterally toward the ventricular apex, descending near the tip of the older ventricular lead.

A 12‐lead ECG revealed atrial and ventricular paced complexes with a monophasic R wave in lead V1 and right axis deviation, an unusual finding as the expected pattern during pacing should be a left bundle branch morphology (Fig. 2). The postimplantation X‐ray revealed a right lateral position of the atrial lead with the new ventricular lead descending toward the apex (Fig. 3).

**Figure 4:**
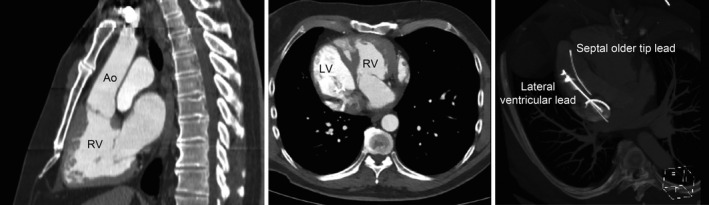
A chest CT angiogram revealed atrioventricular and ventriculoarterial discordance with the anatomic left ventricle (LV) in a “pulmonary” position and the anatomic right ventricle (RV) in a “systemic” position. The new ventricular lead was in a lateral position. Ao refers to the aorta.

In postimplantation of the new pacemaker, a transthoracic echocardiogram was performed. We observed a switch of the two ventricles: The ventricular lead was visualized inside a subpulmonary anatomic left ventricle, connected to a right atrium. The aorta arose from an anatomic right ventricle presenting a moderator band and a tricuspid valve slightly lower than the mitral valve, with the right ventricle connected to an anterior left atrium. While the subaortic right ventricle was hypertrophic, displaying dilatation and global hypokinesis, with a mild tricuspid regurgitation, the function of the subpulmonary left ventricle was normal. A chest computed tomography (CT) angiogram confirmed the incidental detection of a CCTGA and dextrocardia. Surprisingly, there were no other congenital malformations. Computed tomography and echocardiography confirmed a right lateral position of the new ventricular lead and a fracture of the older VDD lead, the tip being localized in a septal position, with a distal part of 55 mm (Fig. 4). This lead has been left in place. Follow‐up 2 months after implantation of the new leads was without any problems. Thereafter, the patient has unfortunately been lost for follow‐up visits.

## Discussion

Congenitally corrected transposition of the great arteries combines atrioventricular and ventriculoarterial discordance. The atria are connected to the opposite ventricle (right atrium to a left ventricle via a mitral valve, and a left atrium to a right ventricle via a tricuspid valve), and the ventricles are connected to the discordant great arteries (anatomic right ventricle to the aorta, and an anatomic left ventricle to the pulmonary artery). If there are no other malformations, blood flows remain separated, systemic flow passing through an anatomic right ventricle, and pulmonic flow through an anatomic left ventricle. Subsequently, the isolated form of this malformation leads to few symptoms and is often diagnosed in adulthood, when cardiac exams are performed for other reasons.

Dextrocardia occurs with normal abdominal situs in 20% of cases of CCTGA 7. In a small case series regarding pacemaker implantation in patients with dextrocardia, all three patients with situs solitus had CCTGA 5. In our patient, the presence of a situs solitus and dextrocardia should probably have raised the suspicion of CCTGA earlier.

Twenty years previously, prior to the implantation of the first VDD pacemaker, a transthoracic echocardiogram had been performed, identifying correctly the dextrocardia, but failing to recognize the precise origin of the great arteries due to the unusual position of the ventricles and a poor echogenicity. There was a doubt about the origin of the pulmonary artery, which appeared similar to the aortic one. No other complementary imaging techniques were performed at that time. Unfortunately, the patient could not be followed closely afterward. The doubt raised later, after visualization of the ventricular lead in a morphologically left ventricle and a dilated, hypertrophied, and hypokinetic right ventricle during a technically difficult echocardiogram. Afterward, a cardiac CT had been performed, confirming the diagnosis of CCTGA.

In CCTGA, the position of the AV node is abnormal, with elongation, fibrosis in its proximal part 3, and displacement to an anterior/superior position within the right atrium 3, 6. When complete heart block is present, the escape QRS complexes have a narrow aspect because conduction is blocked in the proximal part of the node 4, 8. The escape rate is often faster than 50 bpm and is rarely symptomatic 6. In our case, the second‐degree AV block had a junctional escape rate of 36 bpm, but nevertheless well tolerated.

The absence of Q waves in V5–V6 was in accordance with CCTGA. In such patients, the activation of the septum occurs in the opposite direction to that of a normal heart, because the ventricular conduction system is inverted 3, 7. Therefore, Q waves appear in the right precordial leads and are absent in the left precordial leads.

In patients with CCTGA and dextrocardia, a good knowledge of the anatomy seems important prior to implanting pacemaker leads. Indeed, positioning of the ventricular lead can be difficult because the ventricles are rotated to the right, thus increasing the complexity of pacemaker lead implantation. In such situations, fluoroscopic orientation can also be challenging. Jayaprakash Shenthar proposed the use of venous cineangiograms to guide ventricular lead implantation, allowing better differentiation of a lateral position from a septal one, and thus helping to achieve a better pacing site 5. Unfortunately, due to the known dextrocardia, no further attention was paid to the position of the ventricular lead (Fig. 4).

In patients with CCTGA, septal pacing may be associated with a paradoxical motion of the interventricular septum which may cause an aggravation of the systemic right ventricular tricuspid valve regurgitation. Systemic right ventricular function can worsen over time, resulting in congestive heart failure and decreased life expectancy 9. In our patient, the ventricular lead was by chance in contact with the lateral ventricular wall (Fig. 4).

Clear indications for resynchronization therapy (biventricular pacing) in adults with congenital cardiac disease are missing. Hofferberth et al. performed a retrospective analysis of all patients with CCTGA implanted with epicardial pacemakers. Of the 42 patients (79%) who underwent univentricular pacing, 22 (52%) developed a significant systemic ventricular dysfunction. In contrast, the 11 patients (21%) who received from the outset a biventricular pacemaker all maintained a preserved systemic ventricular function. This observation supports the idea that all patients with CCTGA and a complete heart block should be implanted from the outset with a biventricular pacemaker and epicardial leads, at least for those who require concomitant surgical procedures to correct associated defects 10. As said before, ventricular arrhythmias are rare in these patients, defibrillator implantation is recommended only after a cardiac arrest or after an episode of hemodynamically significant or sustained ventricular tachycardia (class IIA, level of evidence C) 1.

In our patient, even after implantation of the new ventricular lead, tricuspid regurgitation remained mild, maybe due to pacing of the free lateral wall of the subpulmonary left ventricle. This case could be used as evidence to argue in favor of subpulmonary left ventricular free wall pacing rather than septal pacing for this kind of congenital defect, in order to preserve systemic right ventricular function. On the last follow‐up visit, the patient was asymptomatic, and we did not notice any deterioration of the systemic right ventricle function.

## Conclusions

Presence of dextrocardia associated with situs solitus should raise the suspicion of CCTGA. Pacemaker implantation is complex and challenging in such patients with congenital cardiac diseases. Additional techniques such as echocardiography, venous cineangiograms, and CT imaging should ideally be used to guide atrial and ventricular lead placement. In these patients, the preferable site for positioning the ventricular lead seems theoretically to be the left lateral wall.

## Conflict of Interest

The authors declare that they have no conflict of interest. The current analysis received no grants from any funding agency.

## Consent for Publication

Informed written consent was obtained from the patient.

## Authorship

AV: wrote the manuscript, collected the data, and performed the data interpretation and literature research; FD: reviewed the manuscript for conception and final editing; SS: cared for the patient (echocardiography) and reviewed the manuscript; MD: cared for the patient (CT scans). DB and DR: cared for the patient and contributed to the coordination and final editing of the manuscript.
